# Baseline vs. on-treatment heart failure with preserved ejection fraction (HFpEF) in a real world cardio-oncology clinic: observational analysis of cancer therapy-related cardiovascular toxicity incidence and cancer treatment implications

**DOI:** 10.3389/fcvm.2026.1816293

**Published:** 2026-06-09

**Authors:** Berlinde von Kemp, Xavier Galloo, Bram Roosens, Bart Neyns, Rik Schots, Mark De Ridder, Bernard Cosyns

**Affiliations:** 1 Centrum Hart- en Vaatziekten (CHVZ)—Cardiology Department, Universitair Ziekenhuis Brussel, Vrije Universiteit Brussel, Brussels, Belgium; 2 Oncology Department, Universitair Ziekenhuis Brussel, Vrije Universiteit Brussel, Brussels, Belgium; 3 Haematology Department, Universitair Ziekenhuis Brussel, Vrije Universiteit Brussel, Brussels, Belgium; 4 Radiotherapy Department, Universitair Ziekenhuis Brussel, Vrije Universiteit Brussel, Brussels, Belgium

**Keywords:** cancer therapy-related cardiac dysfunction, cardio-oncology, cardiotoxicity, heart failure, HFpEF, risk stratification

## Abstract

**Background and aims:**

Heart failure with preserved ejection fraction (HFpEF) prevalence increases, but in cancer patients undergoing potentially cardiotoxic treatments, HFpEF is not included in baseline risk stratification, nor in cancer therapy-related cardiac dysfunction (CTRCD) definitions. Data on HFpEF in this population are scarce. We described baseline HFpEF prevalence in cancer patients and compared incidence of cancer therapy-related cardiovascular toxicity (CTR-CVT), CTRCD and HFpEF events (new HFpEF diagnosis or decompensation of pre-existing HFpEF) and mortality in this subgroup compared to patients without pre-existing HF and to patients with pre-existing HF(m)rEF. Secondly, we investigated the incidence of HFpEF events and CTR-CVT after cancer therapy initiation, identifying predictors for developing HFpEF events.

**Methods and results:**

This retrospective analysis included 665 patients (54.1% female, mean age 62.1 years), of whom 36 (5.4%) had known HFpEF prior to cancer therapy initiation. Compared to patients without HF, pre-existing HFpEF implied higher mortality (27.8% vs. 12.8%, *p* = 0.011), numerically comparable to mortality in pre-existing HF(m)rEF (26.8%), with death occurring significantly earlier in pre-existing HFrEF, driving the mortality signal. Though no difference in CTR-CVT incidence was observed, pre-existing HFpEF predisposed to more HFpEF events (41.6%, *p* < 0.001) and less CTRCD (2.8%). Independent of baseline characteristics, 96/665 patients (14.4%) developed HFpEF events, of whom 47.9% had pre-existing CVD yet only 15/96 (15.6%) had a HFpEF diagnosis. Cancer therapy required adaptation in 12/96 patients (12.5%) but no mortality difference was observed in this subpopulation. Older age, female sex, arterial hypertension and previous arrhythmias predicted HFpEF events in multivariate analysis.

**Conclusion:**

Pre-existing HFpEF carries a significant morbidity and mortality risk, where pre-existing HFpEF identifies a high-risk phenotype with elevated crude mortality, but the independent mortality signal is driven by HFrEF. HFpEF events are common and may have important cancer treatment (and therefore prognostic) implications, despite not being formally included in the definition of “CTRCD” in current guidelines. In patients developing HFpEF events, HFA-ICOS baseline risk stratification proformas suggested a low to intermediate CTR-CVT risk in most patients, possibly reflecting an underestimation of this risk and encouraging further optimization of current risk stratification tools.

## Graphical Abstract

**Figure:**
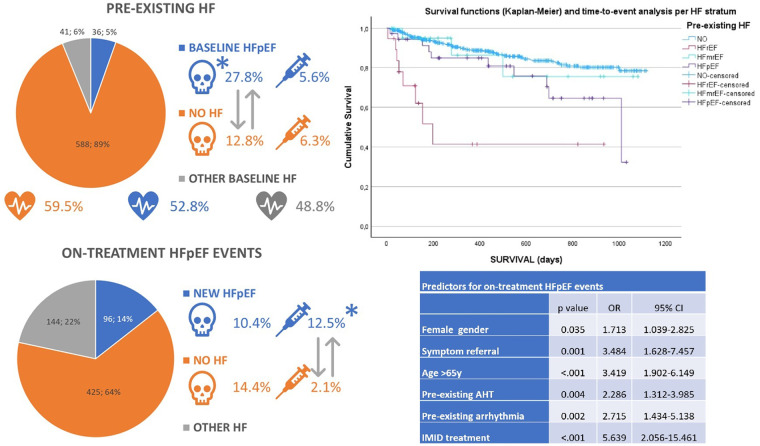
Prevalence of pre-existing HFpEF and incidence of on-treatment HFpEF events in a real-world cardio-oncology outpatient clinic and impact on outcomes. AHT, arterial hypertension; CI, confidence interval; CVD, cardiovascular disease; HF, heart failure; HFpEF, heart failure with preserved ejection fraction; IMID, immunomodulatory multiple myeloma treatment; OR, odds ratio. Icons: skull = mortality, syringe = treatment adaptation, heart = cancer therapy-related cardiovascular toxicity.

## 1 Introduction

Over the past years, the prevalence of heart failure with preserved ejection fraction (HFpEF) has seen a steady increase in the general population, due to heightened clinical awareness, as well as due to the development of improved diagnostic tools and algorithms such as the H2FPEF score and the HFA-PEFF algorithm (1–3). Furthermore, the availability of guideline-recommended treatment options in HFpEF such as sodium-glucose cotransporter 2-inhibitors (SGLT2i), adds important therapeutic implications to this diagnosis (1, 4–6). Left untreated, the prognosis of HFpEF is poor, matching that of some cancers (1, 7).

Conversely, ever evolving cancer treatments also contribute to improved survival in cancer patients, resulting in more cancer-free life years during which cancer survivors are at risk of developing cardiovascular disease (CVD) and heart failure (HF) in particular (8–12). Furthermore, cancer and CVD share common risk factors and common disease pathways (1, 8, 13, 14). Patients undergoing potentially cardiotoxic cancer therapies are therefore recommended to undergo baseline cardiovascular (CV) evaluation prior to cancer treatment initiation in order to perform risk stratification for cancer therapy-related cardiovascular toxicity (CTR-CVT), as well as on-treatment surveillance and long-term follow-up after treatment completion to allow for a timely diagnosis of incident cardiotoxic manifestations (12, 15). However, pre-existing HFpEF is not singularly included as a distinct risk factor for CTR-CVT in most baseline risk stratification proformas [as opposed to heart failure with reduced ejection fraction (HFrEF) and mildly reduced ejection fraction (HFmrEF)] (12, 15). The definition of cancer therapy-related cardiac dysfunction (CTRCD) heavily relies on alterations in left ventricular ejection fraction (LVEF) and global longitudinal strain (GLS), with an additional role for cardiac biomarkers (12, 15).

Therefore, HFpEF often remains overlooked as a manifestation of CTR-CVT, despite dyspnea being a common symptom in cancer patients. Data on HFpEF in cancer patients, however, are scarce, often limited to long-term data or very specific patient populations, and HFpEF is usually not evaluated as an individual study outcome (16–18). The impact of pre-existing HFpEF in patients undergoing (potentially cardiotoxic) cancer therapies is poorly studied, and current guidelines do not provide any guidance on the management of HFpEF decompensations in patients undergoing active cancer therapy.

Furthermore, a growing body of evidence supports a bidirectional interaction between malignancy and HF, with common underlying pathophysiological mechanisms and disease pathways, as well as shared risk factors (cardio-oncology and “reverse cardio-oncology”) (19). Recent research has also demonstrated the additional value of established risk scores (such as the Framinghan Risk Score) by their association with incident cancer as well as HF, expanding its clinical applicability beyond atherosclerotic CVD risk stratification, specifically in cardio-oncology (20). This complex interplay between causative mechanisms and risk factors for both cancer and HF warrants a integrative bidirectional assessment of risks and outcomes in order to improve comprehension and management of long-term risks in this particular population (20).

## 2 Materials and methods

### 2.1 Study objectives

In this study, we evaluated the prevalence of HFpEF at baseline and the incidence of HFpEF events throughout treatment in cancer patients. Firstly, we described the prevalence of baseline, pre-existing HFpEF. In this HFpEF subcohort, we investigated the incidence of CTR-CVT, HF events (CTRCD or HFpEF decompensation) and mortality (primary outcome), and we compared these to cancer patients without pre-existing HF as well as to patients with pre-existing HF(m)rEF (co-primary outcome).

Secondly, in the whole population, we specifically investigated the incidence of HFpEF events (new HFpEF diagnosis or decompensation of pre-existing HFpEF) after cancer treatment initiation (secondary outcomes). We described the patient characteristics as well as CV outcomes of this population in comparison to patients who didn't develop any CTRCD as well as to patients who developed “guideline-defined” CTRCD. Finally, we aimed to identify potential predictors for the development of HFpEF events in cancer patients as well as the impact of pre-existing HF subtypes on mortality in cancer patients.

### 2.2 Study design and patient selection

We performed a monocentric, retrospective, observational analysis on a real-world population of consecutive cancer patients referred to the cardio-oncology outpatient clinic at Universitair Ziekenhuis Brussel between March 17, 2021 and March 17, 2024. These patients were referred by their treating cancer physician (oncologist or haematologist), either for baseline risk stratification, for on-treatment surveillance, for long-term follow-up or for the development of new symptoms suggestive of CVD. The only exclusion criterion was age <18 years at the moment of first consultation.

### 2.3 Study procedures

Considering the retrospective, observational nature of this study, procedures were not prespecified. At the cardio-oncology outpatient clinic, all patients underwent comprehensive standard-of-care workup and follow-up in accordance with current guidelines, consisting of clinical assessment, electrocardiogram, transthoracic echocardiography and biomarker testing (when appropriate and available) (10, 12, 15). Further diagnostic procedures were performed at the discretion of the treating cardiologist. HFpEF diagnosis was made, and events were adjudicated by the treating cardiologist according to current diagnostic criteria as stated in the 2021 European Society of Cardiology (ESC) Guidelines on Acute and Chronic Heart Failure: symptoms and signs of HF, LVEF ≥ 50% and objective evidence of cardiac structural and/or functional abnormalities consistent with the presence of left ventricular (LV) diastolic dysfunction or raised LV filling pressures, including raised natriuretic peptides (6). The H2FPEF score was calculated to further consolidate HFpEF probability, with a score of ≥6/9 indicating a high (>90% probability) of HFpEF (2).

In this study, CTR-CVT and CTRCD were diagnosed as defined by the 2022 ESC Guidelines on cardio-oncology (12). In this document, “CTR-CVT” is considered the umbrella term for all different types of cardiotoxicities, including, among others, “CTRCD”, which is itself defined by a predefined, significant deterioration of LVEF, GLS or cardiac biomarkers as compared to the baseline values (12). For this study, we defined “HFpEF events” as new HFpEF diagnosis or cardiac decompensation requiring hospital admission or outpatient diuretic treatment initiation or intensification in patients with normal LVEF. Furthermore, we defined “HF events” as umbrella term for CTRCD and HFpEF events.

Echocardiographic parameters used for diagnostic evaluation of HFpEF included LVEF (≥50%), left atrial volume index (>34 mL/m^2^), E/e′ ratio at rest (>9), pulmonary artery systolic pressure (>35 mmHg) and tricuspid regurgitation velocity at rest (>2.8 m/s) (6). When available, N-terminal pro-brain natriuretic peptide (NT pro-BNP) values were taken into account (>125 ng/L in sinus rhythm, >365 ng/L in atrial fibrillation) (3).

Comprehensive echocardiography was performed and interpreted by an experienced cardiologist following standard recommendations, using GE Vivid E9 XDClear and GE software (GE Healthcare, Solingen, Germany, Viewpoint version 6.12). Lab analysis of NT pro-BNP values was performed using Cobas 8000 e801 electrochemiluminescence immunoassay (Roche Diagnostics, using Elecsys MODULAR ANALYTICS E170 analysers.

### 2.4 Outcomes

The primary outcome of this study was the prevalence of baseline, pre-existing HFpEF in this cancer patient population as well as the incidence of CTR-CVT, CTRCD, HFpEF events and mortality in this HFpEF subgroup. As co-primary endpoint, we compared these events in patients with HFpEF to patients without pre-existing HF and to cancer patients with pre-existing HF with (mildly) reduced ejection fraction [HF(m)rEF].

The secondary outcomes were the incidence of HFpEF events after cancer treatment initiation. We described patient characteristics and cardiovascular outcomes of this population and we compared these to patients who developed CTRCD as well as to patients who did not develop CTRCD. Finally, we aimed to identify potential predictors for the development of HFpEF events in cancer patients and we studied the impact of pre-existing HF per subtype on mortality outcomes.

### 2.5 Statistical analysis

Categorical variables were expressed as absolute and relative frequencies, continuous variables as mean ± standard deviation. Comparisons of categorical variables were made using Pearson's chi-squared *χ*^2^ test or Fisher's exact test as appropriate. For continuous variables, comparisons were made using independent samples Student's *t*-test. A two-tailed probability value <0.05 was deemed statistically significant. Factors predicting the incidence of on-treatment HFpEF events were identified by uni- and multivariable logistic regression. The final models were determined by a conditional forward selection approach. Odds ratio (OR) and confidence intervals (CI) were reported. Factors impacting mortality were investigated using consecutive uni- and multivariable Cox regression, with adjustment for potential confounders. The final model was determined by conditional forward selection approach. Survival data were analysed using log rank testing and visualised in a Kaplan–Meier curve. Statistical analysis was performed using IBM SPSS Statistics (SPSS v31, Chicago, IL, USA).

## 3 Results

### 3.1 Patient demographics

We included 665 consecutive patients with a mean follow-up of 528 (±333) days (median follow-up 485 days, interquartile range 220–842 days). Baseline patient characteristics and detailed CV and oncologic demographics are presented in Table 1. In the studied cohort, a female predominance was observed (*n* = 365; 64.1%), with a relatively young age at inclusion (62.1 years). Most patients were referred by oncologists (*n* = 445; 66.9%) for symptoms suggestive of CVD (*n* = 289; 43.5%), and 21.7% (*n* = 144) were referred for baseline risk stratification.

**Table 1:** Patient characteristics.

General demographics	*n* (%)
Male—no. (%)	305 (45.9%)
Referral indication	*n* (%)
Baseline evaluation	144 (21.7%)
Surveillance on-treatment	232 (34.9%)
Symptoms	289 (43.5%)
Age at intake—year	62.1 (±15.3)
Age at diagnosis—year	57.8 (±16.2)
Age >65 years—no. (%)	330 (49.6%)
Age >80 years—no. (%)	70 (10.5%)
Referring physician	*n* (%)
Oncologist	445 (66.9%)
Haematologist	176 (26.5%)
Other: surgeon/radiotherapist/cardiologist	44 (6.6%)

Cardiovascular demographics	*n* (%)
BMI at intake—kg/m^2^	26.5 (±5.1)
Pre-existing cardiovascular disease—no. (%)	176 (26.5%)
Pre-existing CAD—no. (%)	68 (10.2%)
Pre-existing HF—no. (%)	77 (11.6%)
HFrEF	19 (2.9%)
HFmrEF	22 (3.3%)
HFpEF	36 (5.4%)
Diastolic dysfunction/cardiometabolic type	18 (50.0%)
Atrial fibrillation-type	12 (33.3%)
Valvular type	2 (5.6%)
Hypertrophic/restrictive type	4 (11.1%)
Pre-existing arrhythmia—no. (%)	69 (10.4%)
Pre-existing PAD—no. (%)	32 (4.8%)
Pre-existing cerebrovascular disease—no. (%)	25 (3.8%)
Pre-existing arterial hypertension (AHT)—no. (%)	341 (51.3%)
Pre-existing hypercholesterolaemia—no. (%)	421 (66.3%)
Pre-existing diabetes—no. (%)	129 (19.4%)
Current smoker—no. (%)	62 (9.3%)
Smoking history—no. (%)	229 (34.4%)

Cancer demographics	
Cancer type	*n* (%)
Breast cancer	188 (28.3%)
Prostate cancer	57 (8.6%)
Lung cancer	42 (6.3%)
Colorectal/other GI cancer	51 (7.7%)
Bladder/other GU cancer	15 (2.3%)
Melanoma	67 (10.1%)
ENT cancer	4 (0.6%)
Gynaecological cancer	17 (2.6%)
CNS cancer	5 (0.8%)
Non-Hodgkin's lymphoma	53 (8.0%)
Hodgkin's lymphoma	17 (2.6%)
Acute leukemia	36 (5.4%)
Chronic leukemia	19 (2.9%)
Multiple myeloma	23 (3.5%)
Other non-haematological malignancy	37 (5.6%)
Other haematological malignancy	34 (5.1%)
St. IV disease—no. (%)	298 (44.8%)
First cancer—no. (%)	583 (87.7%)
Cancer treatment	*n* (%)
Anthracycline therapy	195 (29.3%)
Anti-HER2 based therapy	99 (14.9%)
Fluoropyrimidines	93 (14.0%)
Immune checkpoint inhibition	125 (18.8%)
RAF/MEK inhibition	47 (7.1%)
Angiogenesis inhibition	71 (10.7%)
BCR/ABL inhibition	17 (2.6%)
IMID therapy	25 (3.8%)
Proteasome inhibition	21 (3.2%)
Haematopoietic stem cell transplantation	62 (9.3%)
Chest radiotherapy	189 (28.4%)
Male endocrine therapy	60 (9.0%)
Female endocrine therapy	127 (19.1%)

AHT, arterial hypertension; BCR/ABL, breakpoint cluster region/Abelson murine leukemia (Philadelphia chromosome); BMI, body mass index; CAD, coronary artery disease; CNS, central nervous system; ENT, ear-nose-throat; GI, gastro-intestinal; GU, genito-urinary; HER2, human epidermal growth factor receptor-2; HF, heart failure; HFmrEF, heart failure with mildly reduced ejection fraction; HFpEF, heart failure with preserved ejection fraction; HFrEF, heart failure with reduced ejection fraction; IMID, immunomodulatory agent; RAF/MEK, rapidly accelerated fibrosarcoma/mitogen-activated protein kinase; TKI, tyrosine kinase inhibitor.

Pre-existing CVD was present in 26.5% (*n* = 176), most commonly HF (*n* = 77; 11.6%), arrhythmias (*n* = 69; 10.4%) and coronary artery disease (CAD) (*n* = 69; 10.2%). Cardiovascular risk factors were common, with arterial hypertension (AHT) in 51.3% (*n* = 341), hypercholesterolaemia in 66.3% (*n* = 421), type II diabetes in 19.4% (*n* = 129) and active or previous tobacco use in 43.7% (*n* = 291).

Breast cancer was the most common cancer type (*n* = 188; 28.3%), followed by malignant melanoma (*n* = 67; 10.1%) and prostate cancer (*n* = 57; 8.6%). Cancer was metastatic in 44.8% (*n* = 298) and most were first cancers (*n* = 583; 87.7%). Anthracyclines were most frequently used (*n* = 195; 29.3%) followed by chest radiotherapy (*n* = 189; 28.4%) and female endocrine therapy (*n* = 127; 19.1%). Immune checkpoint inhibitors were used in 18.8% (*n* = 125).

### 3.2 Primary and co-primary outcomes: prevalence of baseline HFpEF vs. no HF and vs. HF(m)rEF and outcomes

Pre-existing HF (i.e., combined HFrEF, HFmrEF and HFpEF) was the most common CV comorbid condition, present in 11.6% of all patients (*n* = 77). Pre-existing HFpEF was present in 36/77 (46.8% of all pre-existing HF, or 5.4% of the entire population) with cardiometabolic diastolic dysfunction (“garden variety”) as most common etiology (50% of pre-existing HFpEF cases), closely followed by atrial fibrillation (33.3% of pre-existing HFpEF cases). The mean H2FPEF score was 5.117 in pre-existing HFpEF. For comparison, HFmrEF was present in 22/77 (28.6% of all pre-existing HF, 3.3% of the entire population) and HFrEF in 19/77 (24.7% of all pre-existing HF, 2.9% of the entire population) (Table 2). The remaining 588 patients had no known pre-existing HF.

**Table 2:** Clinical characteristics and outcomes of patients with pre-existing HFpEF vs. pre-existing other HF vs. patients without pre-existing HF.

	No pre-existing HF (*n* = 588)	*p*	Pre-existing HFpEF (*n* = 36)	Pre-existing HF(m)rEF (*n* = 41)	*p*
General characteristics					
Male gender	252 (42.9%)	0.032	22 (61.1%)	31 (75.6%)	0.171
Referral indication—no. (%)					
Baseline evaluation	124 (21.1%)*	0.578	9 (25.0%)	11 (26.8%)*	0.033
Surveillance on-treatment	210 (35.7%)	0.772	12 (33.3%)	10 (24.4%)	0.751
Symptoms	254 (43.2%)	0.857	15 (41.7%)	20 (48.8%)	0.391
Age at intake—year	60.6 ± 15.2*	<.001	73.5 ± 11.7	73.8 ± 9.3	0.924
Age at diagnosis—year	56.1 ± 16.0*	<.001	71.2 ± 12.1	70.7 ± 9.8	0.671
Age >65 years—no. (%)	263 (44.7%)*	<.001	31 (86.1%)	36 (87.8%)*	0.049
Age >80 years—no. (%)	50 (8.5%)*	<.001	11 (30.6%)	9 (22.0%)	0.738
Cardiovascular demographics					
BMI at intake—kg/m^2^	26.2 ± 5.0*	<.001	30.4 ± 6.0	27.7 ± 4.8*	0.086
Pre-existing cardiovascular disease—no. (%)	100 (17.0%)*	<.001	36 (100%)	40 (97.6%)	n/a
Pre-existing CAD—no. (%)	37 (6.3%)*	<.001	10 (27.8%)	21 (51.2%)*	0.036
Pre-existing arrhythmia—no. (%)	35 (6.0%)*	<.001	16 (44.4%)	18 (43.9%)	0.962
Pre-existing arterial hypertension (AHT)—no. (%)	280 (47.6%)*	<.001	28 (77.8%)	33 (80.5%)	0.770
Pre-existing hypercholesterolaemia—no. (%)	358 (60.9%)*	0.043	28 (77.8%)	35 (85.4%)	0.389
Pre-existing diabetes—no. (%)	98 (16.7%)*	<.001	17 (47.2%)	14 (34.1%)	0.243
Current/previous smoker—no. (%)	247 (42.0%)*	0.539	17 (47.2%)	27 (65.9%)	0.099
BB use	–	–	28 (77.8%)	32 (78.0%)	0.977
RAS inhibitor use	–	–	20 (55.6%)	34 (82.9%)*	0.009
MRA use	–	–	18 (50.0%)	29 (70.7%)	0.064
SGLT2 inhibitor use	–	–	8 (22.2%)	24 (58.5%)*	0.001
Loop diuretic use	–	–	14 (38.9%)	19 (46.3%)	0.512
NT pro-BNP—ng/L	–	–	2,950 ± 5,054	1,269 ± 1,247	0.091
H2FPEF score (/9)—mean	–	–	5.117	–	–
H2FPEF score (/9)—median	–	–	5	–	–
Cancer demographics					
Cancer type—no. (%)					
Breast cancer	175 (29.8%)	0.335	8 (22.2%)	5 (12.2%)	0.241
Prostate cancer	41 (7.0%)*	0.032	6 (16.7%)	10 (24.4%)	0.405
Lung cancer	35 (6.0%)	0.562	3 (8.3%)	4 (9.8%)	0.828
Colorectal/other GI cancer	42 (7.1%)	0.789	3 (8.3%)	6 (14.6%)	0.391
Bladder/other GU cancer	12 (2.0%)	0.764	1 (2.8%)	2 (4.9%)	0.635
Melanoma	61 (10.4%)	0.695	3 (8.3%)	3 (7.3%)	0.868
ENT cancer	2 (0.3%)*	0.04	1 (2.8%)	1 (2.4%)	0.926
Gynaecological cancer	15 (2.6%)	0.933	1 (2.8%)	1 (2.4%)	0.926
CNS cancer	4 (0.7%)	0.171	1 (2.8%)	0 (0%)	n/a
Non-Hodgkin's lymphoma	49 (8.3%)	0.555	2 (5.6%)	2 (4.9%)	0.894
Hodgkin's lymphoma	16 (2.7%)	0.984	1 (2.8%)	0 (0%)	n/a
Acute leukemia	33 (5.6%)	0.467	1 (2.8%)	2 (4.9%)	0.635
Chronic leukemia	18 (3.1%)	n/a	0 (0%)	1 (2.4%)	n/a
Multiple myeloma	22 (3.7%)	0.766	1 (2.8%)	0 (0%)	n/a
Other non-haematological malignancy	33 (5.6%)	0.467	1 (2.8%)	3 (7.3%)	0.370
Other haematological malignancy	30 (5.1%)	0.4	3 (8.3%)	1 (2.4%)	0.868
St. IV disease—no. (%)	258 (43.9%)	0.795	15 (41.7%)	25 (61.0%)	0.091
First cancer—no. (%)	516 (87.8%)	0.436	30 (83.3%)	37 (90.2%)	0.368
Cardiotoxicity incidence					
CTR-CVT incidence	350 (59.5%)	0.424	19 (52.8%)	20 (48.8%)	0.726
HF events incidence	210 (35.7%)	0.29	16 (44.4%)	14 (34.1%)	0.355
CTRCD incidence	133 (22.6%)*	0.004	1 (2.8%)	10 (24.4%)*	0.006
HFpEF events incidence	77 (13.1%)*	<.001	15 (41.6%)	4 (9.8%)*	0.001
CAD incidence	71 (12.1%)	0.237	2 (5.6%)	1 (2.4%)	0.481
AHT incidence	49 (8.3%)	0.562	4 (11.1%)	4 (9.8%)	0.846
Arrhythmia incidence	67 (11.4%)	0.572	3 (8.3%)	3 (7.3%)	0.868
Treatment implications and outcomes					
CPT initiation	41 (7.0%)	0.745	2 (5.6%)	4 (9.8%)	0.493
Cancer treatment alteration (pause/interruption)	37 (6.3%)	0.859	2 (5.6%)	4 (9.8%)	0.493
Death	75 (12.8%)*	0.011	10 (27.8%)	11 (26.8%)	0.926
Time until death (mean, days)	284.3 ± 235.4	0.182	396.5 ± 331.9	HFrEF 85.1 ± 66.1*	0.019
				HFmrEF 281.3 ± 216.0	0.589

“HFpEF events” denote new HFpEF diagnosis or (sub-)acute decompensation requiring hospital admission or diuretic initiation / intensification. “HF events” denote CTRCD + HFpEF events. In HF(m)rEF, HFpEF events may occur if the pre-existing HF had recovered prior to cancer treatment initiation. “CPT” denotes the initiation of beta-blocking agents and/or angiotensin-converting enzyme inhibitors from a cardioprotective point of view.

AHT, arterial hypertension; BB, beta-blocking agent; BMI, body mass index; CAD, coronary artery disease; CNS, central nervous system; CPT, cardioprotective treatment; CTR-CVT, cancer treatment-related cardiovascular toxicity; CTRCD, cancer treatment-related cardiac dysfunction; ENT, ear-nose-throat; GI, gastro-intestinal; GU, genito-urinary; HF, heart failure; HFpEF, heart failure with preserved ejection fraction; MRA, mineralocorticosteroid receptor antagonist; NT pro-BNP, N-terminal pro-brain natriuretic peptide; RAS, renin-angiotensin-aldosterone-system; SGLT2, sodium-glucose-like cotransporter 2.

‘*’ denotes a statistically significant difference.

**Figure 1:**
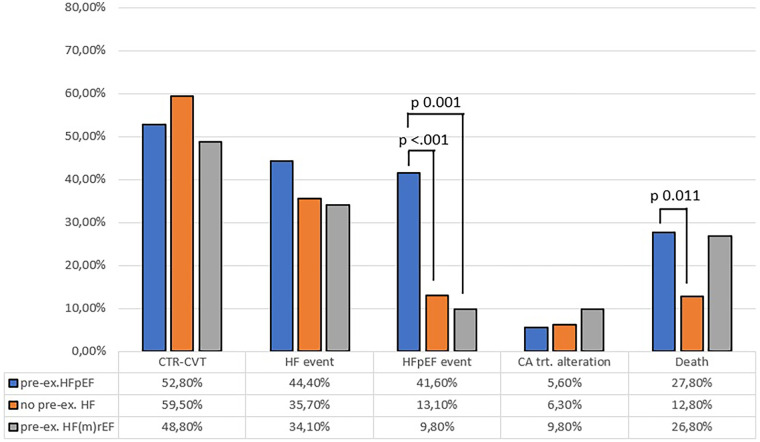
Outcomes in pre-existing HFpEF vs. no HF & vs. pre-existing HF(m)rEF. Upon comparison with patients without pre-existing HF (orange bars), and with patients with pre-existing HF(m)rEF (grey bars), we observed in patients with pre-existing HFpEF significantly more HFpEF events (i.e., HFpEF decompensation requiring diuretic intensification and/or hospital admission). We observed a similar mortality in patients with pre-existing HFpEF as in patients with pre-existing HF(m)rEF, thus indicating the high-risk profile of these patients, particularly compared to patients without pre-existing HF (and also a numerically normal left ventricular ejection fraction). No significant differences were observed for the incidence of CTR-CVT or CTRCD, or for cancer treatment alterations. CA trt., cancer treatment; CTRCD, cancer therapy-related cardiac dysfunction; CTR-CVT, cancer therapy-related cardiovascular toxicity; HF, heart failure; HFpEF, heart failure with preserved ejection fraction; HF**(**m**)**rEF, heart failure with (mildly) reduced ejection fraction; pre-ex., pre-existing.

In pre-existing HFpEF, CTR-CVT developed in 52.8% (*n* = 19), HF events in 44.4% (*n* = 16), CTRCD in 2.8% (*n* = 1) and 27.8% (*n* = 10) died—all deaths in this subgroup were cancer-related (Figure 1).

By comparison, in patients without pre-existing HF (*n* = 588), CTR-CVT developed in 59.5% (*n* = 350, NS), HF events in 35.7% (*n* = 210, NS), CTRCD in 22.6% (*n* = 133, *p* = 0.004) and 12.8% died (*n* = 75, *p* = 0.011). In other patients with pre-existing HF [i.e., HF(m)rEF; *n* = 41], CTR-CVT developed in 48.8% (*n* = 20, NS), HF events in 34.1% (*n* = 14, NS), CTRCD in 24.4% (*n* = 10, *p* = 0.006) and 26.8% died (*n* = 11, NS). Of the 11 deaths in patients with pre-existing HF, three were CVD-related and eight were cancer-related: all CVD-related deaths occurred in patients with pre-existing HFrEF developing worsening HF or arrhythmias. Specifically in patients with pre-existing HFpEF, significantly more HFpEF events were observed (41.6% vs. 13.1% in patients without pre-existing HF, *p* < 0.001).

**Figure 2:**
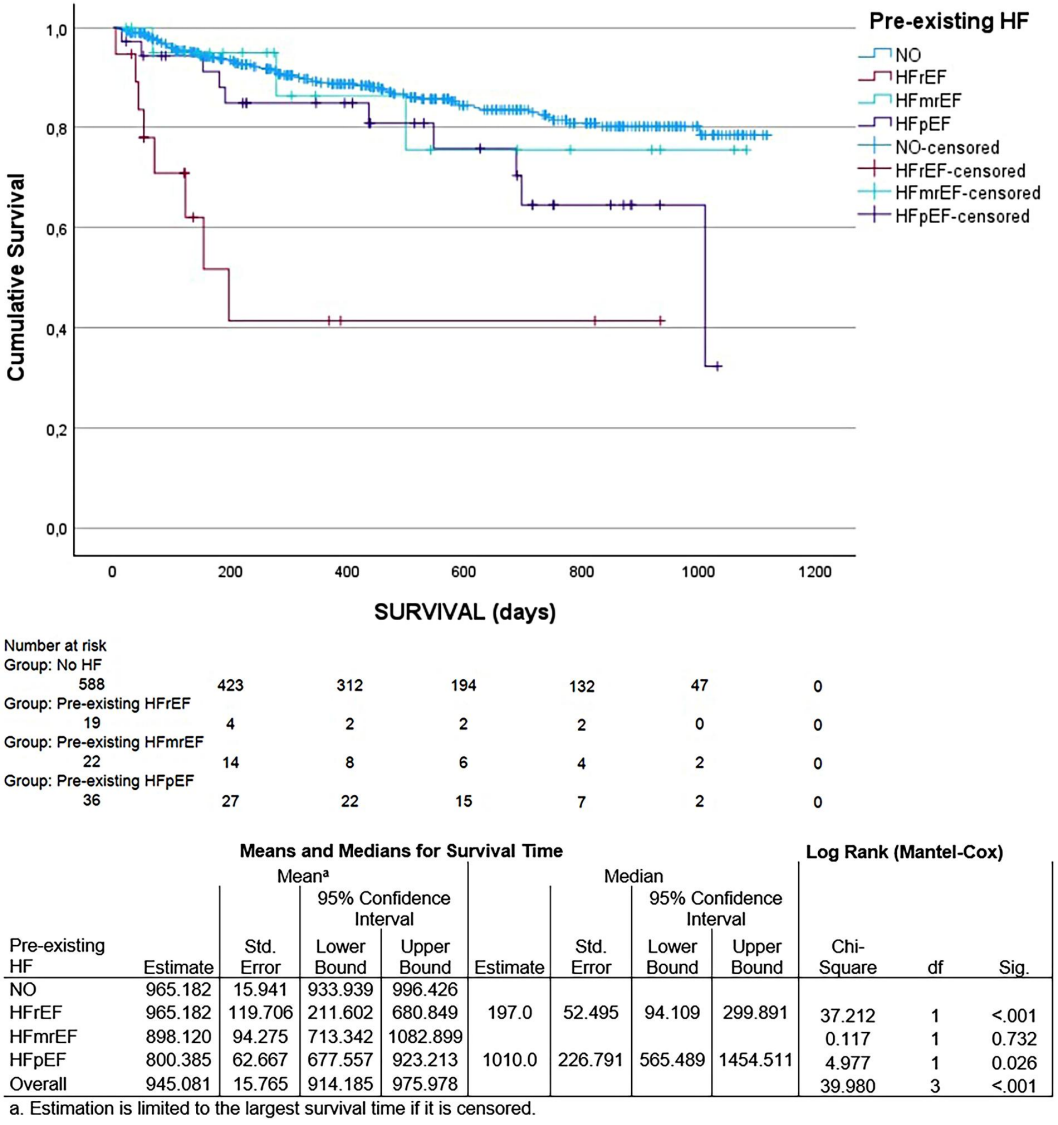
Kaplan–Meier curve for time-to-event analysis (death) per HF stratum. Time-to-event analysis for mortality, stratified for pre-existing HF subtypes. With a caveat of lower absolute event rates, our data showed significantly shorter time-to-event rates for death in patients with pre-existing HFrEF (red line) compared to patients with pre-existing HFmrEF (green line), pre-existing HFpEF (dark blue line) and those without pre-existing HF (light blue line). HF, heart failure; HFmrEF, heart failure with mildly reduced ejection fraction; HFpEF, heart failure with preserved ejection fraction; HFrEF, heart failure with reduced ejection fraction.

Time-to-event analysis for mortality showed significantly earlier mortality in patients with pre-existing HFrEF compared to pre-existing HFpEF (85.1 vs. 396.5 days, *p* = 0.019) (Figure 2). For pre-existing HFmrEF and for patients without pre-existing HF, no significant differences were observed.

### 3.3 Secondary outcomes: HFpEF events in cancer patients

Throughout follow-up, HF events developed in 36.1% of the entire population (*n* = 240). Among these 240 HF events, 96 were HFpEF events, representing 14.4% of the entire population and 40% of all HF events (Table 3). Of these HFpEF events, 68/96 (70.8%) were of the cardiometabolic diastolic dysfunction HFpEF type, and 19/96 (19.8%) were atrial fibrillation-type HFpEF. The mean H2FPEF score was 5.128. Of all 96 HFpEF events, 9 (9.4%) occurred in inpatient setting, and 87 (90.6%) in outpatient context. Of the 144 remaining HF events (i.e., CTRCD), 32 cases were severe CTRCD (HFrEF) (4.8% of the entire population, 13.4% of all HF events), 56 cases were moderate CTRCD (HFmrEF) (8.4% of the entire population, 23.3% of all HF events) and 56 cases were isolated GLS deterioration (8.4% of the entire population, 23.3% of all HF events).

**Table 3:** Patients developing HFpEF events vs. patients developing CTRCD vs. patients without CTRCD.

	No CTRCD (*n* = 425)	*p*	HFpEF events (*n* = 96)	CTRCD (*n* = 144)	*p*
General characteristics					
Male gender	208 (48.9%)	0.097	38 (39.6%)	59 (41.0%)	0.830
Referral indication—no. (%)					
Baseline evaluation	104 (24.5%)*	0.003	10 (10.4%)	30 (20.8%)*	0.034
Surveillance on-treatment	147 (34.6%)	0.225	27 (28.1%)	58 (40.3%)	0.054
Symptoms	174 (40.9%)*	<.001	59 (61.5%)	56 (38.9%)*	<.001
Age at intake—year	61.0 ± 15.5*	<.001	72.3 ± 10.5	58.6 ± 14.6*	<.001
Age at diagnosis—year	57.0 ± 16.4*	<.001	67.1 ± 12.7	54.1 ± 15.5*	<.001
Age >65 years—no. (%)	196 (46.1%)*	<.001	77 (80.2%)	57 (39.6%)*	<.001
Age >80 years—no. (%)	41 (9.6%)*	<.001	23 (24.0%)	6 (4.2%)*	<.001
Cardiovascular demographics					
BMI at intake—kg/m^2^	26.2 ± 4.9*	<.001	28.5 ± 6.1	26.2 ± 4.8	0.768
Pre-existing cardiovascular disease—no. (%)	103 (24.2%)*	<.001	46 (47.9%)	27 (18.8%)*	<.001
Pre-existing HFpEF—no. (%)	20 (4.7%)*	<.001	15 (15.6%)	1 (0.7%)*	<.001
Pre-existing CAD—no. (%)	46 (10.8%)	0.907	10 (10.4%)	12 (8.3%)	0.584
Pre-existing arrhythmia—no. (%)	36 (8.5%)*	<.001	24 (25.0%)	9 (6.3%)*	<.001
Pre-existing arterial hypertension (AHT)—no. (%)	203 (47.8%)*	<.001	73 (76.0%)	65 (45.1%)*	<.001
Pre-existing hypercholesterolaemia—no. (%)	259 (60.9%)*	0.029	74 (77.1%)	88 (61.1%)*	0.010
Pre-existing diabetes—no. (%)	83 (19.5%)	0.155	25 (26.0%)	21 (14.6%)*	0.027
Current/previous smoker—no. (%)	191 (44.9%)	0.442	39 (40.6%)	61 (42.4%)	0.789
BB use	–	–	52 (54.2%)	51 (35.4%)	0.512
RAS-inhibitor use	–	–	43 (44.8%)	57 (39.6%)	0.082
MRA use	–	–	30 (31.3%)	29 (20.1%)*	0.025
SGLT2-inhibitor use	–	–	12 (12.5%)	16 (11.1%)	0.743
Loop diuretic use	–	–	29 (30.2%)	20 (13.9%)*	0.002
NT pro-BNP—ng/L	–	–	2,042 ± 4,693	3,208 ± 9,138	0.285
H2FPEF score (/9)—mean	–	–	5.128	–	–
H2FPEF score (/9)—median	–	–	5	–	–
Cancer demographics					
Cancer type—no. (%)					
Breast cancer	107 (25.2%)	0.103	32 (33.3%)	49 (34.0%)	0.911
Prostate cancer	40 (9.4%)	0.227	13 (13.5%)	4 (2.8%)*	0.001
Lung cancer	28 (6.6%)	0.904	6 (6.3%)	8 (5.6%)	0.822
Colorectal/other GI cancer	37 (8.7%)	0.256	5 (5.2%)	9 (6.3%)	0.735
Bladder/other GU cancer	13 (3.1%)	0.606	2 (2.1%)	0 (0%)	n/a
Melanoma	47 (11.1%)*	0.017	3 (3.1%)	17 (11.8%)	0.150
ENT cancer	3 (0.7%)*	n/a	0 (0%)	1 (0.7%)	n/a
Gynaecological cancer	11 (2.6%)	0.769	3 (3.1%)	3 (2.1%)	0.613
CNS cancer	2 (0.5%)	0.504	1 (1.0%)	2 (1.4%)	0.813
Non-Hodgkin's lymphoma	29 (6.8%)	0.563	5 (5.2%)	19 (13.2%)*	0.043
Hodgkin's lymphoma	13 (3.1%)*	<.001	0 (0%)	4 (2.8%)	n/a
Acute leukemia	17 (4.0%)	0.366	2 (2.1%)	17 (11.8%)*	0.006
Chronic leukemia	16 (3.8%)	0.415	2 (2.1%)	1 (0.7%)	0.343
Multiple myeloma	8 (1.9%)*	<.001	11 (11.5%)	4 (2.8%)*	0.006
Other non-haematological malignancy	25 (5.9%)	0.603	7 (7.3%)	5 (3.5%)	0.184
Other haematological malignancy	29 (6.8%)	0.334	4 (4.2%)	1 (0.7%)	0.065
St. IV disease—no. (%)	194 (45.6%)	0.557	47 (49.0%)	57 (39.6%)	0.151
First cancer—no. (%)	378 (88.9%)	0.212	81 (84.4%)	124 (86.1%)	0.709
Cancer treatment type—no. (%)					
Anthracyclines	105 (24.7%)	0.215	18 (18.8%)	72 (50.0%)*	<.001
Anti-HER2 based therapy	53 (12.5%)	0.577	10 (10.4%)	36 (25.0%)*	0.005
Fluoropyrimidines	48 (11.3%)	0.089	17 (17.7%)	28 (19.4%)	0.736
Immune checkpoint inhibitor(s)	82 (19.3%)	0.187	13 (13.5%)	30 (20.8%)	0.149
RAF/MEK-inhibitors	33 (7.8%)*	0.045	2 (2.1%)	12 (8.3%)*	0.043
Angiogenesis-inhibitors	41 (9.6%)	0.819	10 (10.4%)	20 (13.9%)	0.426
BCR-ABL tyrosine kinase inhibitors	12 (2.8%)	0.873	3 (3.1%)	2 (1.4%)	0.356
Immunomodulatory drugs	10 (2.4%)*	<.001	11 (11.5%)	4 (2.8%)*	0.006
Proteasome inhibitors	10 (2.4%)*	0.004	8 (8.3%)	3 (2.1%)*	0.023
Hematopoeitic stem cell transplantation	35 (8.2%)	0.314	5 (5.2%)	22 (15.3%)*	0.016
Chest/mediastinal radiotherapy	104 (24.5%)	0.913	24 (25.0%)	61 (42.4%)*	0.006
Hormonotherapy (male)	43 (10.1%)	0.328	13 (13.5%)	4 (2.8%)*	0.001
Hormonotherapy (female)	76 (17.9%)	0.364	21 (21.9%)	30 (20.8%)	0.847
Cardiotoxic events					
CTR-CVT incidence	149 (35.1%)	n/a	96 (100%)	144 (100%)	1.0
HFpEF event incidence	0 (0%)	n/a	96 (100%)	0 (0%)	n/a
CAD incidence	37 (8.7%)*	0.009	17 (17.8%)	20 (13.9%)	0.422
AHT incidence	37 (8.7%)	0.835	9 (9.4%)	11 (7.6%)	0.634
Arrhythmia incidence	34 (8.0%)*	<.001	19 (19.8%)	20 (13.9%)	0.225
Treatment implications and outcomes					
CPT initiation	5 (1.2%)*	<.001	7 (7.3%)	35 (24.3%)*	<.001
Cancer treatment alteration (pause/interruption)	9 (2.1%)*	<.001	12 (12.5%)	22 (15.3%)	0.545
Death	61 (14.4%)	0.310	10 (10.4%)	25 (17.4%)	0.135

“HFpEF events” denote new HFpEF diagnosis or (sub-)acute decompensation requiring hospital admission or diuretic initiation/intensification. CPT denotes the initiation of beta-blocking agents and/or angiotensin-converting enzyme inhibitors from a cardioprotective point of view. AHT, arterial hypertension; BB, beta-blocking agent; BCR/ABL, breakpoint cluster region/Abelson murine leukemia (Philadelphia chromosome); BMI, body mass index; CAD, coronary artery disease; CNS, central nervous system; CPT, cardioprotective treatment; CTR-CVT, cancer treatment-related cardiovascular toxicity; CTRCD, cancer treatment-related cardiac dysfunction; ENT, ear-nose-throat; GI, gastro-intestinal; GU, genito-urinary; HER2, human epidermal growth factor receptor-2; HF, heart failure; HFpEF, heart failure with preserved ejection fraction; IMID, immunomodulatory agent; MRA, mineralocorticosteroid receptor antagonist; NT pro-BNP, N-terminal pro-brain natriuretic peptide; PAD, peripheral arterial disease; RAF/MEK, rapidly accelerated fibrosarcoma/mitogen-activated protein kinase; RAS, renin-angiotensin-aldosterone-system; SGLT2, sodium-glucose-like cotransporter 2; TKI, tyrosine kinase inhibitor.

‘*’ denotes a statistically significant difference.

#### 3.3.1 Demographics of cancer patients with HFpEF

##### 3.3.1.1 Cancer patients with baseline, pre-existing HFpEF

When comparing cancer patients with pre-existing HFpEF (*n* = 36) to patients without pre-existing HFpEF (*n* = 588) (Table 2), the majority were male (61.1% vs. 42.9%, *p* = 0.032) and >65 years (86.1% vs. 44.7%, *p* < 0.001). Pre-existing CAD was present in 27.8% (*n* = 10; vs. 6.3%, *p* < 0.001) and pre-existing arrhythmias in 44.4% (*n* = 16; vs. 6.0%, *p* < 0.001) of patients with pre-existing HFpEF. The burden of all CV risk factors (AHT, hypercholesterolaemia, diabetes, smoking) was significantly higher in pre-existing HFpEF, as was the mean body mass index (BMI) (30.4 vs. 26.2 kg/m^2^, *p* < 0.001). Breast cancer was the most common cancer diagnosis (22.2%; *n* = 8) followed by prostate cancer (16.7%; *n* = 6). Most cancers were first malignancies (83.3%, *n* = 30), a small minority had metastatic disease (41.7%, *n* = 15).

Compared to patients with pre-existing HF(m)rEF (*n* = 41), patients with pre-existing HFpEF had been less commonly referred for baseline assessment prior to cancer therapy (25.0% vs. 26.8%, *p* = 0.033). Patients with pre-existing HFpEF showed no significant differences to patients with pre-existing HF(m)rEF in terms of pre-existing CV risk factors and in terms of other CV comorbidities, except for a significantly lower rate of pre-existing CAD (27.8% vs. 51.2%, *p* = 0.036). In pre-existing HF(m)rEF, we observed the following prescription rates of guideline-directed medical therapy: beta-blocking agents (BB) in 78.0%, renin-angiotensin-aldosterone-system (RAS)-inhibiting agents in 82.9%, mineralocorticosteroid receptor antagonists (MRA) in 70.7%, SGLT2-inhibitors in 58.5%. Loop diuretics were prescribed in 46.3%. Compared to pre-existing HF(m)rEF, we observed significantly lower prescription rates of RAS-inhibition (55.6% vs. 82.9%, *p* = 0.009) and SGLT2-inhibition (22.2% vs. 58.5%, *p* = 0.001) in pre-existing HFpEF patients. Natriuretic peptide levels were high in both groups, with even higher mean values in pre-existing HFpEF, but these differences were not statistically significant.

Baseline HFA-ICOS risk stratification was available in 17/36 patients with pre-existing HFpEF (47.2%): zero patients classified as “low-risk”, 2/17 (11.8%) patients as “intermediate risk”, 10/17 (58.8%) as “high-risk” and 5/17 (29.4%) as “very high-risk”, with the median in the “high CTR-CVT risk” stratum—mainly driven by comorbidies, CV risk factors and age. For the remaining 19 patients, treatment consisted of a regimen for which no risk stratification proforma exists to date.

##### 3.3.1.2 Cancer patients developing HFpEF events

Compared to cancer patients who did not develop CTRCD, those who developed HFpEF events (*n* = 96) after cancer therapy initiation (Table 3), were predominantly female (60.4%, *n* = 58) and significantly older: 80.2% were >65 years and 24.0% were >80 years (vs. 51.1% and 9.6%, respectively, *p* < 0.001 for both). Mean age at intake was 72.3 ± 10.5 years (vs. 61.0 ± 15.5 years, *p* < 0.001). Most patients were referred for new symptoms (61.5% vs. 40.9%, *p* < 0.001), and only few had been initially referred for baseline risk stratification (10.4% vs. 24.5%, *p* < 0.001).

Patients who developed HFpEF events had significantly more pre-existing CVD (47.9% vs. 24.2%, *p* < 0.001), most commonly under the form of pre-existing arrhythmias (25.0% vs. 8.5%, *p* < 0.001), pre-existing HFpEF (15.6% vs. 4.7%, *p* < 0.001) and pre-existing CAD (10.4% vs. 10.8%, NS). The CV risk factor burden was significantly higher in patients developing new HFpEF events, with significantly more AHT (76.0% vs. 47.8%, *p* < 0.001), hypercholesterolaemia (77.1% vs. 60.9%, *p* = 0.01) and a higher BMI (mean BMI 28.5 ± 6.1 vs. 26.2 ± 4.9 kg/m^2^, *p* < 0.001).

Most HFpEF events occurred in breast cancer (33.3%, *n* = 32), prostate cancer (13.5%, *n* = 13) and multiple myeloma (11.5%, *n* = 11). Metastatic disease was present in 49.0% (*n* = 47) and most cancers were first malignancies (84.4%, *n* = 81). Specifically for patients with multiple myeloma developing HFpEF events (*n* = 11), AL amyloid cardiomyopathy was considered: serum free light chain testing was performed in all 11 patients (100%), cardiac magnetic resonance imaging in 8/11 (72.7%) and bone scintigraphy in 4/11 (36.4%).

Patients developing HFpEF events were significantly more frequently treated with immunomodulatory multiple myeloma therapies (11.5% vs. 2.4%, *p* < 0.001) and proteasome inhibitors (8.3% vs. 2.4%, *p* = 0.007). For anthracyclines, anti-HER2 based therapies, chest radiotherapy and endocrine therapies (male and female), no significant differences were observed.

In comparison to patients who developed CTRCD (*n* = 144), patients who developed HFpEF events had been less commonly referred for baseline assessment prior to cancer therapy initiation (10.4% vs. 20.8%, *p* = 0.034) and far more frequently for onset of new symptoms (61.5% vs. 38.9%, *p* < 0.001). Patients developing HFpEF events were significantly older, with 80.2% aged 65 years and 24.0% aged >80 years (vs. 39.6% and 4.2%, respectively, *p* < 0.001 for both). Pre-existing CVD was more common in patients developing HFpEF events (47.9% vs. 18.8%, *p* < 0.001), especially pre-existing HFpEF (15.6% vs. 0.7%, *p* < 0.001) and pre-existing arrhythmia (25.0% vs. 6.3%, *p* < 0.001). Furthermore, patients developing HFpEF events had significantly more CV risk factors (AHT, hypercholesterolaemia and diabetes). CTRCD was seen more commonly than HFpEF events in patients with non-Hodgkin's lymphoma (13.2% vs. 5.2%, *p* = 0.043) and in acute leukemia (11.8% vs. 2.1%, *p* = 0.006), whereas in multiple myeloma, HFpEF events were more common (11.5% vs. 2.8%, *p* = 0.006). Significantly more HFpEF events developed in patients undergoing immunomodulatory treatments (11.5% vs. 2.8%, *p* = 0.006), proteasome inhibitor treatment (8.3% vs. 2.1%, *p* = 0.023) and male endocrine therapies (13.5% vs. 2.8%, *p* = 0.001). Conversely, significantly more CTRCD developed in those undergoing anthracycline therapy (50.0% vs. 18.8%, *p* < 0.001), anti-HER2 based therapy (25.0% vs. 10.4%, *p* = 0.005), RAF/MEK-inhibitor therapy (8.3% vs. 2.1%, *p* = 0.043), hematopoietic stem cell transplantation (15.3% vs. 5.2%, *p* = 0.016) and chest or mediastinal radiotherapy (42.4% vs. 25.0%, *p* = 0.006). In patients developing CTRCD, we observed the following prescription rates of cardiovascular agents at inclusion: BB in 35.4%, RAS-inhibiting agents in 39.6%, MRA in 20.1%, SGLT2-inhibitors in 11.1%. Loop diuretics were prescribed in 13.9%. Compared to patients developing CTRCD, we observed significantly higher baseline use of MRA (31.3% vs. 20.1%, *p* = 0.025) and loop diuretics (30.2% vs. 13.9%, *p* = 0.002) in patients developing HFpEF events. Natriuretic peptide levels were high in both groups but did not differ significantly between patients developing CTRCD and those developing HFpEF events. Baseline HFA-ICOS risk stratification was available in 65/96 patients developing HFpEF events (67.7%), with a median in the “intermediate CTR-CVT risk” stratum. In 21/65 patients (32.3%) CTR-CVT risk classified as “low-risk”, in 24/65 (36.9%) as “intermediate risk”, in 19/65 (29.2%) as “high-risk” and in one (1.5%) as “very high-risk”. For the remaining 31 patients, treatment consisted of a regimen for which no risk stratification proforma exists to date.

**Figure 3:**
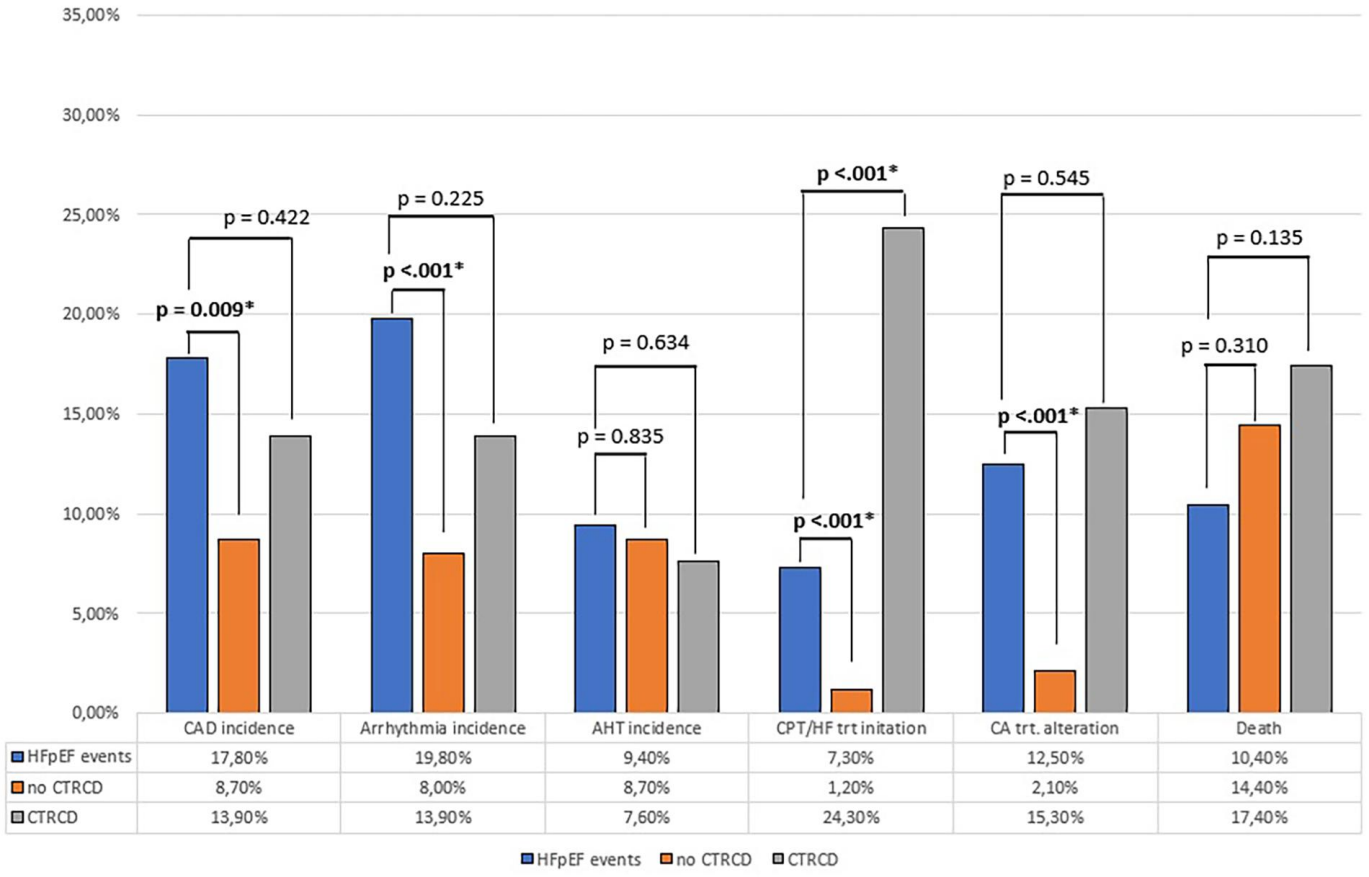
Outcomes in patients developing HFpEF events vs. no CTRCD vs. CTRCD. When comparing to patients without CTRCD, patients who developed HFpEF events after cancer therapy initiation developed significantly more CAD and arrhythmias (comparable to patients developing CTRCD). Furthermore, patients developing HFpEF events required significantly more cancer treatment alterations than patients without CTRCD, again comparable to patients with CTRCD. Cardioprotective treatment or heart failure treatment was initiated significantly less often than in CTRCD but significantly more often than in patients without CTRCD. However, this seemed to imply no significant mortality difference throughout the follow-up period. AHT, arterial hypertension; CAD, coronary artery disease; CA trt., cancer treatment; CPT, cardioprotective treatment; CTRCD, cancer therapy-related cardiac dysfunction; HFpEF, heart failure with preserved ejection fraction; HF trt, heart failure treatment.

In terms of outcomes (Figure 3), patients with new HFpEF events also developed more new CAD (17.8% vs. 8.7%, *p* = 0.02) and more new arrhythmias (19.8% vs. 8.0%, *p* = 0.02) than those without CTRCD, without significant differences compared to those who developed CTRCD. Cardioprotective therapies were initiated in 7.3% (vs. 1.2% in those without CTRCD, *p* < 0.001) and cancer treatment had to be discontinued (temporarily or definitively) significantly more frequently (12.5% vs. 2.1%, *p* < 0.001) compared to patients without CTRCD (Supplementary Table A). In patients developing CTRCD, cardioprotective treatment (i.e., heart failure therapy in these cases) was initiated more commonly than in those developing HFpEF (24.3% vs. 7.3%, *p* < 0.001), yet the amount of cancer treatment interruptions was not significantly different (15.3% vs. 12.5%, *p* = 0.545).

We observed no significant mortality difference (10.4% in patients with HFpEF events vs. 14.4% in those without CTRCD and vs. 17.4% in those with CTRCD). All deaths in patients developing HFpEF events were cancer-related, of the 25 deaths in patients with CTRCD, 19 were cancer-related and 6 were CVD-related.

#### 3.3.2 Risk predictors associated with HFpEF events

In the 96 patients developing HFpEF events, potential predictors associated with the development of these HFpEF events were investigated using consecutive univariable (Table 4) and multivariable logistic regression analysis (Table 5). Independent predictors for the development of HFpEF events in cancer patients were female sex [Odds ratio (OR) for male sex 0.595], symptom referral (OR 3.417), age >65 years (OR 3.406), AHT (OR 2.324), pre-existing arrhythmia (OR 2.77) and immunomodulatory multiple myeloma treatment (OR 5.49). The female-sex effect remained significant independent of age, but lost significance in separate landmark analysis (with landmark time cutoff of 365 days). Nagelkerke's *R*^2^ of the final model was 0.252, with C-statistic of 0.790 for area under the curve in ROC curve analysis, and a Hosmer-Lemeshow goodness-of-fit *p*-value of 0.115 for the final step in the stepwise forward conditional model.

**Table 4:** Univariable binary logistic regression analysis.

	Univariable *p* value	OR	95% CI
General characteristics			
Female sex	0.098	1.463	0.932–2.297
Referral indication—no. (%)			
Baseline evaluation	<.001	0.226	–
Surveillance on-treatment	0.099	1.91	0.886–4.116
Symptoms	<.001	3.526	1.729–7.194
Age >65 years—no. (%)	<.001	4.735	2.767–8.101
Age >80 years—no. (%)	<.001	2.951	1.671–5.211
Cardiovascular demographics			
Pre-existing cardiovascular disease—no. (%)	<.001	2.867	1.814–4.532
Pre-existing HFpEF—no. (%)	<.001	3.682	1.805–7.511
Pre-existing CAD—no. (%)	0.907	0.958	0.465–1.974
Pre-existing arrhythmia—no. (%)	<.001	3.602	2.028–6.397
Pre-existing arterial hypertension (AHT)—no. (%)	<.001	3.471	2.093–5.756
Pre-existing hypercholesterolaemia—no. (%)	0.003	2.156	1.289–3.606
Pre-existing diabetes—no. (%)	0.157	1.451	0.867–2.428
Current smoker—no. (%)	0.681	1.173	0.549–2.508
Previous smoker—no. (%)	0.28	0.763	0.467–1.247
Cancer demographics			
Previous cancer treatment—no. (%)			
Previous anthracycline treatment—no. (%)	0.482	1.513	0.477–4.798
Previous chest radiotherapy—no. (%)	0.181	1.957	0.732–5.231
Current cancer treatment—no. (%)			
Anthracycline treatment—no. (%)	0.216	0.703	0.403–1.229
Anti-HER2 treatment—no. (%)	0.578	0.578	0.399–1.669
Fluoropyrimidine treatment—no (%)	0.089	1.69	0.924–3.092
ICI treatment—no. (%)	0.19	0.655	0.348–1.233
RAF/MEK inhibitor treatment—no. (%)	0.062	0.253	0.060–1.072
Angiogenesis inhibitor treatment—no. (%)	0.819	1.089	0.525–2.259
BCR/ABL TKI treatment—no. (%)	0.873	1.11	0.307–4.013
IMID treatment—no. (%)	<.001	5.371	2.211–13.047
Proteasome inhibitor treatment—no. (%)	0.007	3.773	1.448–9.831
Hematopoietic stem cell transplantation—no. (%)	0.319	0.612	0.233–1.606
Chest radiotherapy—no. (%)	0.913	1.029	0.616–1.717
Male endocrine therapy—no. (%)	0.33	1.391	0.716–2.703
Female endocrine therapy—no. (%)	0.365	1.286	0.746–2.215
Stadium IV disease—no. (%)	0.557	1.142	0.733–1.779
First cancer—no. (%)	0.214	0.671	0.358–1.259

AHT, arterial hypertension; BCR/ABL, breakpoint cluster region/Abelson murine leukemia (Philadelphia chromosome); CAD, coronary artery disease; CNS, central nervous system; CTRCD, cancer treatment-related cardiac dysfunction; ENT, ear-nose-throat; GI, gastro-intestinal; GU, genito-urinary; HER2, human epidermal growth factor receptor-2; HF, heart failure; HFpEF, heart failure with preserved ejection fraction; IMID, immunomodulatory agent; OR, odds ratio; RAF/MEK, rapidly accelerated fibrosarcoma/mitogen-activated protein kinase; TKI, tyrosine kinase inhibitor.

**Table 5:** Multivariable analysis for association of on-treatment HFpEF.

	*p* value	OR	95% CI
Significant risk factors for on-treatment HFpEF events			
Female sex	0.035	1.713	1.039–2.825
Symptom referral	0.001	3.484	1.628–7.457
Age >65 years	<.001	3.419	1.902–6.149
Pre-existing AHT	0.004	2.286	1.312–3.985
Pre-existing arrhythmia	0.002	2.715	1.434–5.138
IMID treatment	<.001	5.639	2.056–15.461

AHT, arterial hypertension; CI, confidence interval; IMID, immunomodulatory agent; OR, odds ratio.

#### 3.3.3 Pre-existing HF subtype and mortality risk

Of the 96 registered deaths, 75 (78.1%) occurred in patients without pre-existing HF, 8 (8.3%) in pre-existing HFrEF, 3 (3.1%) in pre-existing HFmrEF and 10 (10.4%) in pre-existing HFpEF. Using consecutive univariable and multivariable Cox regression analysis (Table 6), only pre-existing HFrEF appeared to be a significant predictor for death (HR 3.889, 95% CI 1.799–8.406), even after adjustment for potential confounders as patient age, cancer type and metastatic disease status.

**Table 6:** Multivariable Cox regression analysis for death per HF stratum.

	Multivariable *p* value	HR	95% CI
Pre-existing HF	0.002	–	–
Pre-existing HFrEF	**<0** **.** **001**	**3** **.** **889**	**1.799–8.406**
Pre-existing HFmrEF	0.878	1.095	0.344–3.490
Pre-existing HFpEF	0.194	0.629	0.313–1.267
Confounding factors	–	–	–
Patient age	0.327	–	–
Cancer type	0.449	–	–
Metastatic disease	0.936	–	–

HF, heart failure; HFmrEF, heart failure with mildly reduced ejection fraction; HFpEF, heart failure with preserved ejection fraction; HFrEF, heart failure with reduced ejection fraction.

Despite numerically comparable mortality in patients with pre-existing HF(m)rEF and HFpEF, only pre-existing HFrEF (values in bold) appears to be a significant driver of survival differences in time-to-event analysis.

Survival data and time-to-event analysis for death across the different strata of pre-existing HF using log rank testing, revealed a significant difference (*χ*^2^ statistic 39.980; df = 3, *p* < 0.001) between strata, primarily driven by pre-existing HFrEF. For pre-existing HFpEF, median survival time was 1,010 days (95% CI 565.5–1,454.5 days), while for pre-existing HFrEF median survival time was 197 days (95% CI 94.1–299.9 days) (Figure 2). Separate analyses for pre-existing HF subtypes vs. no pre-existing HF were consecutively performed. For pre-existing HFrEF vs. no pre-existing HF, time-to-event analysis revealed the most significant difference (*χ*^2^ statistic 37,212; df = 1, *p* < 0.001). For pre-existing HFpEF vs. no pre-existing HF, the time-to-event analysis also confirmed a significant difference (*χ*^2^ statistic 4.997; df = 1, *p* < 0.026). No significant difference was observed for pre-existing HFmrEF, likely owing to the low number of events in this stratum.

## 4 Discussion

This study delivers relevant new insights regarding the clinical impact of HFpEF in cancer patients, both in terms of pre-existing HFpEF and new HFpEF events after cancer treatment initiation, based on real-world data from a dedicated cardio-oncology service. These data may prove to be valuable in optimizing cardiotoxicity risk stratification as well as in correct CTR-CVT diagnosis and possibly even in cancer prognosis. Considering the bidirectional relationship between cancer and CVD, this study uniquely assessed not only the impact of cancer and cancer treatment on CV outcomes (CTR-CVT), but also the impact of pre-existing CVD on cancer-related outcomes and mortality (20).

First and foremost, our findings confirm a significantly higher mortality burden in cancer patients with pre-existing HFpEF compared to patients without pre-existing HF. This highlights the importance of a thorough baseline CV evaluation prior to cancer therapy initiation, which needs to focus on more than only LVEF (which is normal in both groups). Furthermore, we observe numerically comparable mortality in patients with pre-existing HFpEF and in pre-existing HF(m)rEF, despite normal LVEF in pre-existing HFpEF. However, in time-to-event analysis, survival differences were mainly driven by pre-existing HFrEF, and to a lesser extent by pre-existing HFpEF.

Secondly, pre-existing HFpEF implies a high CTR-CVT burden and a high rate of HF events (CTRCD and HFpEF events). Patients with pre-existing HFpEF also show a comparable CV comorbidity and risk factor profile as patients with pre-existing HF(m)rEF.

Third, HFpEF events seem to make up 40% of all HF events that occurred throughout cancer treatment, despite not fitting into the current guideline-defined diagnostic criteria of CTRCD (12). However, these events may have important therapeutic implications for cancer treatment (and prognosis): in 12.5% of patients developing HFpEF events, cancer treatment was discontinued—either temporarily or definitively.

### 4.1 Baseline prevalence of HFpEF in cancer patients

In the general population, HF prevalence is estimated at 1%–2% of adults and increases with age: in outpatient HF patients, the ESC Long-Term Registry reported HFrEF in 60%, HFmrEF in 24% and HFpEF in 16 % (6, 18). However, more recent studies demonstrate increasing HFpEF proportions in the general HF population (1, 21).

Despite increasing awareness of CTR-CVT, CTRCD and the increasing development of dedicated cardio-oncology services, data on pre-existing HF in cancer patients are limited, in particular for HFpEF (17, 19–23). Our data showed pre-existing HF in 11.6% of the studied population (46.8% HFpEF, 28.6% HFmrEF and 24.7% HFrEF), and pre-existing HFpEF in 5.4% of the studied population. Compared to cancer patients without pre-existing HF, those with pre-existing HFpEF were more often male, older, obese, with a higher burden of pre-existing CVD and CV risk factors [comparable to patients with pre-existing HF(m)rEF], with cardiometabolic diastolic dysfunction as most common HFpEF phenotype. These findings are in line with the clinical characteristics of a general (non-cancer) HFpEF population, except for the male sex predominance. As in a general population, in patients with pre-existing HFpEF, breast cancer and prostate cancer were the most commonly encountered cancer types.

Previous studies in in elderly Hodgkin lymphoma patients and diffuse large B-cell lymphoma patients demonstrated a prevalence of pre-existing HF in 13.4% and 13.9%, respectively (24, 25). In lung cancer patients, pre-existing HF was seen in 17.5% (26). For breast cancer patients, a baseline HF prevalence was observed in only 1.5%–4.9%, and in a cohort of hospitalized cancer patients, a comorbid HF diagnosis was registered in 7.2% (27–29). However, none of these studies differentiated between HF subtypes (HFrEF, HFmrEF or HFpEF) and nearly all studies relied on population-based clinical registries or administrative databases for HF diagnostics (24–29). Only one study on immune checkpoint inhibitor therapy included 82 patients with pre-existing HF: of these, 22% (*n* = 18) had HFrEF, 8% (*n* = 7) had HFmrEF and 67% (*n* = 55) had HFpEF (30).

Our data demonstrate a higher prevalence of pre-existing HF than in the breast cancer studies, yet lower than the lymphoma and lung cancer studies, possibly due to the heterogeneity of the studied populations: on average, the breast cancer cohorts were younger and both lymphoma studies were prespecified to analyse older patients, while a comparable age distribution was observed in the lung cancer cohort (24–28). As in many cardio-oncology studies, this heterogeneity complicates generalizability across broader cancer populations (31, 32).

Our findings represent real-world outpatient data in a broad range of cancer types, and is to our knowledge the only study where HF diagnosis is based on actual clinical and echocardiography data (as per current ESC HF Guidelines), rather than on database entries, and one of very few studies to differentiate between the different HF subtypes and HFpEF phenotypes (6). Furthermore, our data offer unique insight into baseline HF therapy prescription rates in cancer patients with pre-existing HF, demonstrating nearly 80% prescription rates of guideline-directed medical therapy in pre-existing HF(m)rEF, with an exception for SGLT2i. These prescription rates are in line with previous prescription data of HF therapy in cancer patients from the Swedish Heart Failure Registry and the National Cancer Register, where patients with HFrEF and previous cancer appeared to be less likely to be treated with MRA and guideline-directed medical therapy for HF, compared to HF patients without cancer (33). Nevertheless, it deserves mentioning that our studied patient population included more patients undergoing active cancer treatment and more recent cancer diagnoses than the population studied in the Swedish Heart Failure Registry and the National Cancer Register, who had a mean time of 9.6 years since last cancer—a relative survival bias may therefore be considered upon comparison of these data (33). The lower rate of SGLT2-inhibitor use in pre-existing HFpEF reflects not only a relative undertreatment of these patients, but mainly the lack of Belgian government-funded reimbursement of SGLT2-inhibitors in HFpEF indication throughout the study inclusion period.

Finally, in the studied population, the prevalence of pre-existing HFpEF seems low, possibly reflecting underdiagnosis. This hypothesis may be further supported by the observation that nearly half of patients who developed HFpEF events throughout cancer treatment, had pre-existing CVD (and may therefore be assumed to have undergone a previous CV evaluation before cancer treatment), as well as by the observation of significantly higher baseline prescription rates of MRA and loop diuretics in patients developing HFpEF events.

### 4.2 Prognostic value of pre-existing HFpEF in cancer patients

To date, we have no knowledge of other studies investigating CTR-CVT, CTRCD and mortality specifically in cancer patients with pre-existing HFpEF. In a general HF population, however, it has been demonstrated that HFpEF patients had a higher burden of noncardiac comorbidities, resulting in poorer functional status and clinical outcomes (34, 35). Our findings suggest a high incidence of CTR-CVT and HF events in cancer patients with pre-existing HFpEF (52.8% and 44.4%, respectively), though this did not differ significantly from patients without pre-existing HF. However, pre-existing HFpEF predisposed to a significantly higher incidence of HFpEF decompensations and less to “classical” CTRCD. This is further supported by the finding that in patients with pre-existing HFpEF, where HFA-ICOS risk stratification was available (47.2% of patients), the baseline risk assessment indeed suggested a high to very high CTR-CVT risk in most patients, mainly driven by age, CV risk factors and comorbid conditions.

We also observed a significantly higher mortality in cancer patients with pre-existing HFpEF compared to cancer patients without pre-existing HF [27.8% vs. 12.8%, *p* = 0.02, hazard ratio (HR) 2.17], where all deaths were cancer-related. Furthermore, this mortality rate was comparable to patients with pre-existing HF(m)rEF (26.8%). Both this mortality difference, as well as the significantly higher rate of HFpEF decompensations and comparable death rates as in pre-existing HF(m)rEF, identifies patients with pre-existing HFpEF as a particularly high-risk cohort despite a numerically normal LVEF. However, time-to-event analysis showed that despite comparable crude mortality rates between pre-existing HFpEF and pre-existing HF(m)rEF, death occurred significantly earlier in the specific subcohort of pre-existing HFrEF (mean time to death 85.1 vs. 396.5 days, median survival time of 197 days in HFrEF). Log rank analysis showed a significant survival difference between patients with pre-existing HF vs. those without pre-existing HF, mainly driven by the mortality in pre-existing HFrEF and to a lesser extent by mortality in pre-existing HFpEF (Figure 2).

Cox regression analysis confirmed the significance of pre-existing HFrEF as risk predictor for mortality, even after adjustments for patient age, cancer type and metastatic disease status. Specifically for mortality, pre-existing HFpEF could not be withheld as an independent risk predictor in Cox regression analysis.

Previous studies indeed report worse cancer-related outcomes in cancer patients with HF compared to those without HF. Currently available data confirm increased CV and lymphoma mortality in elderly Hodgkin's lymphoma (HR 2.57 and 1.25, respectively) and diffuse large B-cell lymphoma patients (HR 1.24 for lymphoma mortality) with pre-existing HF as well as increased mortality (HR 1.85) in lung cancer patients with pre-existing HF (24–26). Comparable increases in cancer mortality were seen in breast cancer patients (HR 2.28) and hospitalized cancer patients (12.2% inpatient mortality in HF patients) (27, 28). However, none of these studies differentiated between HFrEF, HFmrEF and HFpEF.

Our findings indeed confirm that pre-existing HFpEF conveys a worse prognosis in cancer patients and suggests that the findings in selected cancer types, may extend to a more general population of cancer patients. Further specific comparison on CTR-CVT and CTRCD is currently impossible due to lack of other studies specifically focusing on HFpEF patients, highlighting the need for further research in this population.

### 4.3 Incidence and prognosis of HFpEF after cancer treatment initiation

In the entire studied population, our findings show a high incidence of CTR-CVT (58.5%, *n* = 389) and HF events (36.1%, *n* = 240), despite the relatively young age of the studied patients. Of all HF events, 40% were HFpEF events: these represent 14.4% of the entire studied population (compared to 8.4% and 4.8% who developed HFmrEF and HFrEF, respectively). However, we did observe that in patients developing HFpEF events, HFA-ICOS baseline risk stratification had identified a majority of these patients to be at low or intermediate CTR-CVT risk. These observations may suggest a relative underestimation of the risk of HFpEF events using the HFA-ICOS risk stratification proformas, and that further optimization of these risk stratification tools may prove valuable.

In terms of outcomes and prognosis, no significant mortality difference was observed in patients who developed HFpEF events throughout treatment, but we did observe a significantly higher amount of cancer therapy interruptions in these patients (12.5% vs. 2.1%, *p* < 0.001), possibly impacting cancer prognosis on the longer term. Longer follow-up will be required to assess this hypothesis. Furthermore, in this population we also observed significantly more CAD and arrhythmic events, which may further affect CV prognosis but may also impact future cancer treatment choices.

Historically, literature has mainly focused on the development of left ventricular systolic dysfunction (LVSD) by means of a deteriorating LVEF and/or GLS value for the diagnosis of CTRCD (10, 12). Although recently a position statement on right ventricular dysfunction in cancer patients has been published, studies focusing on HFpEF in patients undergoing cancer treatment are scarce and current ESC Guidelines do not explicitly mention HFpEF in the diagnostic criteria for CTRCD (12, 17, 36).

Currently available evidence is mostly limited to breast cancer patients, limiting generalizability to broader cancer populations (37–42). Compared to non-cancer controls, breast cancer survivors appear to have a significantly higher risk of incident HFpEF at long term follow-up (HR 1.22): this risk was confirmed in patients undergoing chemotherapy (HR 1.67) and in patients undergoing chest radiotherapy (HR 1.40) but not in those receiving endocrine therapy (37). However, 10-year cumulative incidence rates were very low (1.2% in overall HF, 0.4% for HFrEF and 0.8% for HFpEF) (37). This increased risk also translates to more HF hospitalizations in breast cancer survivors (6.68% HFpEF vs. 3.96% HFrEF hospitalizations over a 7.2 years follow-up) (38). In older women developing HF after radiotherapy, HFpEF was the predominant HF subtype (64% of HF cases, vs. 10% in HFrEF) on a HF incidence of 6.2 % (39). In a real-world cohort study in young breast cancer patients, a high long-term incidence of CTR-CVT was shown (71.8% over 19.3 years follow-up) as well as HF incidence up to 47%, not differentiating between HF subtypes (40). Echocardiography studies in breast cancer patients suggest that cancer treatment may induce diastolic dysfunction, but the authors consider this finding a potential precursor of guidelines-defined CTRCD, rather than as actual HFpEF (41, 42).

Our findings suggest a high incidence of CTR-CVT, CTRCD and HFpEF events in a real-world, heterogeneous cancer population, and possible CTRCD underestimation in currently available data, which are mainly limited to breast cancer patients (who may have their own particular cardiotoxicity phenotype). Furthermore, our findings underline the importance of considering HFpEF as a genuine cardiotoxic event (occurring 3 times as frequently as HFrEF and almost twice as frequently as HFmrEF) as well as the importance of acknowledging pre-existing CVD in identifying patients at risk of developing HFpEF throughout cancer treatment.

Data on prognostic implications of new-onset HFpEF throughout cancer treatment are even more limited: only one study in breast cancer survivors reports a higher mortality risk (5.65 in hospitalized HFpEF vs. 3.77 in hospitalized HFrEF at 7.2 years follow-up) (38). Our data did not show a significant mortality difference in cancer patients developing HFpEF compared to cancer patients without CTRCD, with the caveat of a shorter follow-up duration. However, in patients who developed HFpEF throughout cancer treatment, we did see significantly more initiation of cardioprotective treatments and particularly significantly more cancer treatment discontinuation (either temporary or definitive). The follow-up duration of our study population did not allow to evaluate the impact of these cancer treatment alterations on cancer therapy completion and cancer outcomes (such as progression-free survival): further research is warranted on this subject. In this context, the conceptual framework of “permissive cardiotoxicity” is highly relevant, accepting and balancing a somewhat increased HF risk to ensure optimal cancer treatment (43, 44). However, data on permissive cardiotoxicity are currently limited to anti-HER2 based therapies in breast cancer patients, excluding moderate and severe LV dysfunction, thus limiting generalizability to a broader cancer population (43, 44).

Furthermore, with the availability of SGLT2i as a guideline-recommended therapy in HFpEF, and the poor prognosis of HFpEF in a general population, competing risk analyses between cancer outcomes and cardiovascular outcomes may prove insightful, as well as the added value of HFpEF treatments in cancer patients.

Concerning cardiovascular outcomes, patients who developed HFpEF also seemed to develop more CAD and arrhythmic events: though none of the deaths in our population were cardiovascular deaths, these events do highlight the particularly high CVD risk of these patients as well as the importance of optimal preventive measures.

### 4.4 Characteristics of patients developing HFpEF after cancer treatment initiation

In our study, patients who developed HFpEF after cancer treatment initiations are more often female, and older than patients without CTRCD. Furthermore, these patients had a significantly higher BMI, and more CV risk factors (AHT, hypercholesterolaemia and diabetes), which is in line with findings in a general HFpEF population (1). Of all patients who developed HFpEF events, 47.9% had pre-existing CVD, yet only 15.6% had a pre-existing HFpEF diagnosis. Nevertheless, pre-existing HFpEF was significantly more common in this population compared to patients without CTRCD, as were pre-existing CAD and pre-existing arrhythmias. This is reflected in the fact that cardiometabolic diastolic dysfunction and atrial fibrillation-related HFpEF where the most commonly observed HFpEF phenotypes in these patients. Strikingly, patients who developed HFpEF were most frequently referred because of new symptoms, and only a minority of these high-risk patients had been referred for baseline risk stratification prior to cancer treatment initiation (despite a high prevalence of pre-existing CVD). This may explain the lower rates of CV treatments (BB, RAS-inhibition, MRA, SGLT2i and loop diuretic use) upon study inclusion in these patients.

We observed most HFpEF events in patients with breast cancer and prostate cancer, mainly due to the high prevalence of these cancers. However, we did observe significantly more multiple myeloma patients who developed HFpEF compared to myeloma patients without CTRCD (11.5% vs. 1.9%)—possibly identifying a subpopulation at risk for underlying (AL) amyloid cardiomyopathy. This highlights the importance of integrating screening investigations for this specific condition in multiple myeloma patients, with an important role for clinical cardiac and extracardiac red flags, multimodality imaging (cardiac magnetic resonance imaging and bone scintigraphy) as well as biomarker testing (free light chain assays). Similarly, significantly more patients treated with immunomodulatory agents and proteasome inhibitors, developed HFpEF, but this difference was not observed for hematopoietic stem cell transplantation.

We identified independent risk predictors for the development of HFpEF throughout cancer treatment in this real-world population, revealing female sex, referral for symptoms, age >65 years, pre-existing AHT, pre-existing arrhythmias and treatment with immunomodulatory agents as independent predictors for the development of HFpEF throughout cancer treatment. The female-sex effect remained significant irrespective of age. Despite observing significantly more HFpEF events in patients with prostate cancer and undergoing treatment with male endocrine therapy, these factors did not appear to be significant independent predictors for HFpEF events. It should be noted, however, that the amount of prostate cancer patients included in this study, was relatively small in comparison to the general prevalence of prostate cancer as most common cancer in men. This may reflect a selection and/or referral bias where the patients referred to cardio-oncology units by their treating oncologist already are a selected group of patients with higher CV risk. Furthermore, this may support the hypothesis that prostate cancer patients are a patient population in whom cardio-oncological assessment and surveillance throughout cancer treatment, is still less systematically performed (as opposed to surveillance in breast cancer patients and in patients undergoing anthracycline-based chemotherapy). Finally, with the increasing use of androgen-receptor targeting agents, future research in this population may warrant a more granular assessment and comparison between “standard” androgen deprivation therapy (i.e., gonadotropin-releasing hormone agonists and antagonists) and anrdrogen-receptor targeting therapies in terms of HFpEF events.

### 4.5 Perspectives

#### 4.5.1 Diagnosis of HFpEF and baseline cardiotoxicity risk prediction

Our study demonstrated a striking difference between the amount of patients with pre-existing HFpEF and patients developing HFpEF events after cancer treatment initiation. Current baseline risk stratification proformas do not explicitly mention HFpEF as an individual risk factor for the development of CTR-CVT. While our findings did not show a significant difference in CTR-CVT and HF events compared to patients without pre-existing HF, we did observe a significantly higher mortality which was comparable to patients with pre-existing HF(m)rEF. This highlights the importance of a comprehensive baseline assessment prior to initiation of cancer therapy, looking beyond a normal LVEF on echocardiography, and the added value of considering HFpEF as a risk factor for CTR-CVT in baseline risk assessment. However, it is worth mentioning that specifically pre-existing HFrEF carries the worst prognosis with significantly earlier mortality.

In patients who develop HFpEF events after cancer treatment initiation, the mortality burden is less obvious, but cancer treatments were significantly more frequently interrupted. Though this didn't seem to affect mortality during this follow-up period, long-term cancer prognosis may be affected by this. Therefore, further investigations and longer follow-up is necessary, and HFpEF events deserve specific attention and recognition as cardiotoxic events. The finding that a majority of patients developing HFpEF events had been initially stratified as “low-risk” or “intermediate-risk” using HFA-ICOS CTR-CVT risk stratification proformas, may suggest a relative underestimation of HFpEF event risk using this tool.

The high prevalence of pre-existing CVD yet the lower prescription rates of CV therapies in patients developing HFpEF throughout cancer treatment may suggest a relative underdiagnosis or at least late diagnosis as well as a relative undertreatment of HFpEF in these patients, thus highlighting both diagnostic and therapeutic opportunities to improve outcomes in this high-risk patient population. Furthermore, in light of potential interactions of HF pharmacotherapy with cancer disease pathways in pre-clinical research, further synchronized oncological and CV clinical research on this particular subject may prove valuable (45). Current research, however, faces particular obstacles in this area: cancer outcomes are often poorly assessed in CV research, and we observe a similar issue of poor reporting of CV outcomes in cancer trials (45–47).

#### 4.5.2 Prediction of development of HFpEF after cancer treatment initiation

Cancer patients who developed HFpEF after cancer treatment initiation had been significantly less frequently referred for baseline assessment prior to cancer therapy, and more commonly for onset of new symptoms. This suggests a potentially missed opportunity for earlier diagnosis and treatment of HFpEF.

We identified female sex, age >65 years, pre-existing hypertension and pre-existing arrhythmias as independent risk predictors for the development of HFpEF during cancer therapy. In these patients, diagnostic tools as the H2FPEF score, the HFA-PEFF algorithm and cardiac biomarkers may prove to be of added value to confirm the diagnosis earlier on. New therapies in HFpEF such as SGLT2-inhibitiors have proven their value in a general HFpEF population, their potential benefit as cardioprotective agent in cancer patients undergoing potentially cardiotoxic treatments, is currently being investigated. Furthermore, considering the observation of more HFpEF events in prostate cancer patients and patients undergoing male endocrine therapies, but without significant predictive signal from the logistic regression analyses, this may prove to be a valuable subject fur further investigations.

### 4.6 Strengths and limitations

This was a single-center, retrospective, observational study, and therefore has some inherent limitations. Since we included consecutive patients referred to the cardio-oncology clinic, this is a heterogeneous population in terms of cancer types and treatment types. Therefore, prospective validation of these findings is required, eventually focusing of specific cancer types or treatment types. Secondly, a selection bias is possible: all included patients were referred by their cancer physician for dedicated cardiovascular assessment. Patients in whom a low cardiotoxicity risk was assumed by their treating oncologist or haematologist, may not have been systematically referred unless upon development of new signs and symptoms suggestive of (new) CVD. Finally, as this is a retrospective observational study, some data are missing: NT pro-BNP measures weren't available in all patients, and in particular the HFA-ICOS risk stratification was only feasible in patients who underwent treatment with one of the cancer treatments for which a proforma was available.

However, this heterogeneity also represents a significant strength of this study: this extensive, real-world, patient population includes a fair amount of patients with pre-existing heart disease, who are otherwise commonly excluded from other trials. Furthermore, this heterogeneity probably gives a more realistic insight into an actual “real-world” cancer population as encountered in oncology and haematology clinics on a daily basis. This study provides a unique insight into CTR-CVT, CTRCD, HFpEF events and mortality risks in a by default high-risk patient cohort, where data from randomized trials are scarce.

## 5 Conclusion

In conclusion, pre-existing baseline HFpEF conveys a high risk of CTR-CVT and implies an important morbidity (HF events, both CTRCD and HFpEF events) and mortality burden, especially compared to patients without pre-existing HF. However, HFpEF still seems underdiagnosed in the global cancer population. By demonstrating a comparable mortality as in pre-existing HF(m)rEF, we underline the importance of considering pre-existing HFpEF in baseline assessment prior to cancer therapy initiation. Pre-existing HFpEF identifies a high-risk phenotype with elevated crude mortality, but the independent mortality signal is driven by HFrEF.

Incident HFpEF events appear to be more common than incident HF(m)rEF after cancer therapy initiation, and these occur most commonly in older women, breast cancer, prostate cancer and multiple myeloma. In 12.5% of patients who developed HFpEF events, cancer treatment was temporarily or definitively interrupted, which may significantly affect cancer prognosis. This impact on cancer therapy management highlights the importance of specifically considering HFpEF events as CTR-CVT. Risk predictors for HFpEF events after cancer therapy initiation are female sex, symptom referral, age >65 years, AHT, pre-existing arrhythmia and immunomodulatory myeloma therapy. Further investigations are required to evaluate whether specific management strategies may improve prognosis in these patients.

## Data Availability

The raw data supporting the conclusions of this article will be made available by the authors, without undue reservation.
