# Supplementary material for: Baseline vs. on-treatment heart failure with preserved ejection fraction (HFpEF) in a real world cardio-oncology clinic: observational analysis of cancer therapy-related cardiovascular toxicity incidence and cancer treatment implications
Source: Front Cardiovasc Med. 2026 Jun 9;13:1816293. doi: 10.3389/fcvm.2026.1816293 (PMC13286771; doi:10.3389/fcvm.2026.1816293)
Supplement: Supplementary file 1 [file Datasheet1.docx]

| **SUPPLEMENTARY TABLE A : Cancer treatment interruptions** | | | | | | | | | | |
| --- | --- | --- | --- | --- | --- | --- | --- | --- | --- | --- |
| **Case nr** | **Sex** | **Age** | **Cancer type** | **Metastatic disease** | **Pre-existing CVD** | **Implicated treatment type** | **Interruption type** | **Resumption status** | **Oncologic outcomes** | **Death** |
| 1 | F | 83 | NSCLC | yes | n/a | entrectinib | definitive stop | no | disease progression +7m | no |
| 2 | F | 66 | cervix cancer | yes | n/a | bevacizumab | definitive stop | no | complete remission | no |
| 3 | M | 52 | NSCLC | yes | n/a | pembrolizumab + pemetrexed | temporary interruption pembrolizumab 2m (pemetrexed continued) | rechallenge after 2m | disease progression | no |
| 4 | M | 61 | multiple myeloma | no | n/a | carfilzomib | definitive stop | no (switch to new treatment regime) | disease progression | no |
| 5 | F | 79 | multiple myeloma | no | n/a | carfilzomib | temporary interruption 6w | yes dose reduction | disease progression | no |
| 6 | F | 73 | breast cancer | yes | n/a | letrozole | definitive stop | no (switch to new treatment regime) | complete remission | no |
| 7 | M | 76 | GIST | no | pre-existing CVD | imatinib | definitive stop | no | complete remission | no |
| 8 | F | 76 | breast cancer | no | HFpEF, SVT | trastuzumab | definitive stop | no | complete remission | no |
| 9 | M | 70 | prostate cancer | yes | Aortic valve stenosis, HFpEF | apalutamide | definitive stop | no | stable disease | no |
| 10 | M | 74 | thyroid cancer | yes | HFpEF, AF, moderate aortic valve stenosis, aortic root dilatation | cabozantinib + cemiplimab | definitive stop cabozantinib (cemiplimab continued) | no | disease progression | yes |
| 11 | F | 84 | breast cancer | no | HFpEF, AF, CAD + VHD (AVRbio + MVRbio) | trastuzumab | temporary interruption (6w) | rechallenge, shortened treatment duration due to recurrent decompensation | complete remission | no |
| 12 | F | 74 | multiple myeloma | no | n/a | lenalidomide + daratumumab | definitive stop | no (switch to new treatment regime) | disease progression | no |

Supplementary table A : Cancer treatment interruptions.

AF = atrial fibrillation, AVRbio = bioprosthetic aortic valve replacement, CAD = coronary artery disease, CVD = cardiovascular disease, GIST = gastro-intestinal stromal tumor, HFpEF = heart failure with preserved ejection fraction, MVRbio = bioprosthetic mitral valve replacement, NSCLC = non-small cell lung cancer, SVT = supraventricular tachycardia, VHD = valvular heart disease
